# Regarding: the role of fat-soluble vitamins A, D, E, and K in osteoporosis: mechanisms, interactions, and evidence

**DOI:** 10.1080/07853890.2025.2573140

**Published:** 2025-10-15

**Authors:** Shuai Chen

**Affiliations:** The Affiliated Yan’ an Hospital of Kunming Medical University, Yunnan Province, China

## To the editor,

We read with great interest the recent review entitled ‘The role of fat-soluble vitamins on bone metabolism and osteoporosis: a literature review’ [1]. This work provides a comprehensive and timely synthesis of the biological pathways, clinical evidence, and emerging concepts linking these vitamins with bone health. Notably, the authors successfully integrated mechanistic insights such as Wnt/β-catenin signalling, γ-carboxylation and ferroptosis with clinical outcomes, while also applying GRADE evaluation to clinical trials, which enhances the reliability of their conclusions. This multidimensional approach highlights the importance of considering fat-soluble vitamins not in isolation but as part of a complex network influencing bone metabolism.

While the review represents a valuable contribution, we believe there are several important limitations that merit further consideration. First, the lack of distinction between dietary intake and supplemental forms may limit clinical applicability. Nutrients obtained from whole foods often act synergistically with other dietary components, whereas supplements deliver isolated compounds in pharmacological doses. For example, vitamin K2 from fermented foods such as natto has demonstrated stronger bone-protective effects compared with pharmacological vitamin K1 [2]. Future work should more explicitly compare natural dietary sources versus supplement interventions, as the metabolic effects and safety profiles may differ substantially.

Second, genetic variability and drug–nutrient interactions were not sufficiently discussed. Polymorphisms in genes such as VDR, GC or VKORC1 can alter absorption, transport, or activity of these vitamins, leading to heterogeneous treatment responses. Moreover, vitamin K supplementation may interact with anticoagulant therapy [3], and vitamin A excess may have toxic skeletal consequences [4]. Incorporating pharmacogenomic insights and medication histories into clinical studies would improve precision in both research and patient care.

Third, the review primarily emphasized bone mineral density (BMD) as a clinical endpoint, but BMD alone is an imperfect surrogate for fracture risk. Functional outcomes such as incident fractures, fall risk, and patient-reported quality of life are of greater clinical relevance [5]. Future trials should extend beyond short-term BMD changes and adopt longer follow-up with fracture events as primary endpoints. Moreover, standardized biomarker panels (e.g. ucOC, P1NP, CTX, lipid peroxidation markers) could provide mechanistic validation and facilitate cross-trial comparability [6].

In conclusion, this review significantly advances our understanding of the interplay between fat-soluble vitamins and bone health. By highlighting both mechanistic novelty and clinical translation, it provides a valuable resource for researchers and clinicians. Addressing the limitations of dietary versus supplemental forms, genetic and pharmacologic modifiers, and clinically meaningful endpoints will strengthen future investigations. We commend the authors for their contribution and hope our reflections may stimulate more rigorous and targeted research to improve prevention and management strategies for osteoporosis.

## Data Availability

Data sharing is not applicable to this article as no data were created or analysed in this study.
